# Pulmonary Hypertension: The Hallmark of Acute COVID-19 Microvascular Angiopathy?

**DOI:** 10.1183/23120541.00389-2022

**Published:** 2023-02-02

**Authors:** Sri Harsha Dintakurti, Sanjana Kamath, Ciara Mahon, Suveer Singh, Bhavin Rawal, Simon P. G. Padley, Anand Devaraj, Laura C. Price, Sujal R. Desai, Tom Semple, Carole A. Ridge

**Affiliations:** 1 Imperial College London, London, UK; 2 Department of Imaging, Royal Brompton Hospital, London, UK; 3 Department of Adult Intensive Care, Royal Brompton Hospital, London, UK; 4 Pulmonary Hypertension Service, Royal Brompton Hospital, London, UK

## Abstract

There have been over 481 million cases of Coronavirus Disease-19 (COVID-19), caused by the SARS-CoV-2 virus worldwide since December 2019 [1]. One of the hallmark features of acute COVID-19 pneumonia is pulmonary vascular involvement, most commonly manifesting as pulmonary artery thrombosis (PAT) [2, 3]. Post-mortem data in ten patients with COVID-19 pneumonia shows their central pulmonary arteries were free of thrombosis but all patients had small, firm thrombi in the peripheral parenchyma [4]. These findings raise the possibility that the CT finding of isolated subsegmental PAT may reflect “the tip of the iceberg”; that small segmental thrombi may reflect downstream *in situ thrombosis* in the microvasculature. In patients with severe COVID-19 pneumonitis, Dual-Energy CTPA (DECTPA) has been used to demonstrate reduced pulmonary perfusion in the absence of any visible central thromboembolism [5, 6], further supporting the view that microscopic PAT is prevalent [6].

This is a PDF-only article. Please click on the PDF link above to read it.

